# Identification of pVHL as a Novel Substrate for Aurora-A in Clear Cell Renal Cell Carcinoma (ccRCC)

**DOI:** 10.1371/journal.pone.0067071

**Published:** 2013-06-13

**Authors:** Benedicte Martin, Franck Chesnel, Jean-Guy Delcros, Florence Jouan, Anne Couturier, Frederic Dugay, Xavier Le Goff, Jean-Jacques Patard, Patricia Fergelot, Cecile Vigneau, Nathalie Rioux-Leclerq, Yannick Arlot-Bonnemains

**Affiliations:** 1 CNRS - UMR 6290 (IGDR) -Université Rennes 1- BIOSIT - Rennes, France; 2 UMR- INSERM U1052/CNRS 5286, Centre de Recherche en Cancérologie de Lyon, Centre Léon Bérard, Lyon, France; 3 CHU Bordeaux, Service de Génétique Moléculaire, Bordeaux, France; 4 AP-HP Hôpital Bicêtre, Service de Chirurgie Urologie, Le Kremlin Bicêtre, France; 5 CHU Pontchaillou, Département de Néphrologie, Rennes, France; 6 CHU Pontchaillou, Département de Pathologie, Rennes, France; Institut de Génétique et Développement de Rennes, France

## Abstract

Clear cell renal cell carcinoma (ccRCC) is the most common histological subtype of kidney cancer and is often characterized by mutations or deletions of the Von Hippel Lindau (VHL) tumour suppressor gene. Aurora gene family members are implicated in proper mitotic progression and spindle checkpoint function and play a crucial role in cancer progression. In the present study, we assessed the expression of Aurora-A in a cohort of 30 ccRCC with fully characterized VHL status (wt/wt or mut/del) and Fuhrman grade. Aurora-A transcript and protein levels were significantly increased in high Fuhrman grade tumours and in VHLwt/wt tumours. These results suggest that Aurora-A and VHL interact in the ccRCC. We demonstrated that the two proteins interact in vivo and identified the Ser72 on the sequence of VHL as the unique site phosphorylated by Aurora-A.

## Introduction

Clear cell renal cell carcinoma (ccRCC) is the most common form of kidney cancer and the yearly number of newly diagnosed cases is steadily increasing. This type of cancer develops in the renal proximal tube and is linked to biallelic inactivation of the von Hippel–Lindau (VHL) tumour suppressor gene. VHL syndrome is an inherited disorder caused by germline point mutations in the *VHL* gene and is characterized by a predisposition to a variety of benign and malignant tumours [1]. Based on the patients’ genotype, VHL syndrome can be divided in four subtypes (1, 2A, 2B and 2C) that are associated with different phenotypes [2] [3] [4]. Only the 1 and 2B subtypes predispose patients to the development of ccRCC. Biallelic inactivation of *VHL* has also been reported in up to 80% of patients with sporadic ccRCC [5]; [6][7];. *VHL* mutations are extremely heterogeneous and distributed all along the gene (promoter, exons and introns). Despite the observation that the reintroduction of wild type *VHL* in a *VHL*-null ccRCC cell line inhibits tumorigenesis [8], the molecular basis of tumour formation and progression upon VHL inactivation remains unclear. However, additional oncogenic events are required for ccRCC formation as clearly evidenced by molecular and genetic approaches [9].

There are three isoforms of the *VHL* gene product (known as pVHL): the full-length pVHL30 protein (213 amino acids) and two smaller isoforms of 160 (pVHL19, due to the use of an internal translational start site) and 172 amino acids (the result of alternative splicing) in length. pVHL is thought to be an E3 ubiquitin ligase within a tetramer complex with Cullin 2 and Elongins B and C that targets hypoxia inducible factor α (HIFα) for polyubiquitylation and proteasome degradation. This implicates pVHL in the regulation of genes involved in cell proliferation and survival and in angiogenesis. In addition, pVHL exerts other functions that are completely independent of its role in HIFα degradation, including regulation of microtubule stabilization, cell cycle progression [8,10] maintenance of appropriate Mad2 protein levels and chromosomal stability [11].

Many protein kinases are key regulators of cell cycle progression and are frequently deregulated in cancer. Among them, the mitotic Aurora-A serine/threonine (Ser/Thr) kinase has been identified as a putative oncogenic protein [12]. Over-expression of Aurora-A protein in HeLa cells promotes the formation of colonies in soft agar and favours growth of tumour xenografts in mouse. However, it remains unclear how Aurora-A over-expression can induce oncogenic transformation. Aurora-A expression is cell-cycle regulated as it is degraded *via* the ubiquitin-proteasome pathway upon mitotic exit [13]. It plays a major role during duplication and separation of centrosomes and also contributes to spindle formation and stability. Aurora-A alterations are correlated with aggressive tumour behaviour such as differentiated tumour grade, invasion and nodal metastasis [14] [15]. Amplification of the *Aurora-A* gene and up-regulation of Aurora-A transcript/protein levels or kinase activity have been observed in a variety of cancers [14] [16] [17]. Aurora-A is often expressed in ccRCC and some studies reported elevated expression levels of Aurora A and Aurora B in advanced stage tumours associated with poor patients’ survival [18] [19]. In addition, abrogation of the mitotic spindle checkpoint has been involved in the progression of ccRCC [19]. Altogether these results suggest that Aurora-A may be implicated in an early event of ccRCC development [20] [21].

To test this hypothesis, we first assessed Aurora-A and pVHL expression in ccRCC surgical samples with known VHL status and then investigated their possible interaction. We show that the Aurora-A transcript and protein levels in ccRCC were correlated with the tumour grade and VHL status. Moreover, we demonstrate that Aurora-A interacts with pVHL and that VHL is phosphorylated by Aurora-A.

## Material and Methods

### Tissue samples

Tumour and matched normal tissue samples were obtained from 30 patients with ccRCC who underwent partial or total nephrectomy between 2002 and 2005. The Ethics Committee of the Medical School and Hospital of Rennes University approved this prospective study and all patients signed informed consents. Upon collection, tissue samples were immediately frozen in liquid nitrogen and stored at -80°C at the Centre de Ressources Biologiques (CRB, Rennes, France) or formalin-fixed and paraffin-embedded. The diagnosis and tumour staging were determined by histopathological analysis. To assess *VHL* status in the collected samples, denaturing high-performance liquid chromatography (DHPLC) was carried out using a WAVE Nucleic Acid Fragment Analysis system (Transgenomic, Glasgow, UK) and DNAsep columns [22]. Aberrant peaks were further analysed by direct sequencing using standard procedures. All mutations were confirmed by PCR analysis and sequencing. Multiplex Ligation-dependent Probe Amplification (MLPA) was used to detect *VHL* deletions as previously described [23]. The wild type status was hereafter defined as VHLwt/wt and the samples deleted and mutated for VHL as VHLmut/del.

### Antibodies

The polyclonal antibodies against Aurora-A and Actin were purchased from Abcam (Paris, France) and Sigma-Aldrich (L’Isle d’Abeau, France), respectively. The anti-pVHL monoclonal antibody was from BD Pharmingen (San Jose, CA). The horseradish peroxidase-conjugated secondary antibodies were from Jackson ImmunoResearch Laboratories (Baltimore, MD).

### Real time PCR analysis

Total RNA was extracted from tumour samples using the Qiagen Qiamp Total RNA kit and then five micrograms of each sample were reverse-transcribed using oligo-dT primers and the M-MLV reverse transcriptase. The resulting cDNAs were used as templates for PCR assays. Primers were: 5’ CTGCATTTCAGGACCTGTTAAGG-3’ (forward) and 5’-AACGCGCTGGGAAGAATTT-3’ (reverse) for human Aurora A; 5’-CTGACTTCAACAGCGACACC-3’ (forward) and 5’-TAGCCAAATTCGTTGTCATACC-3’ (reverse) for human GAPDH (control). Assays were performed in triplicate, using a RotorGene 3000 apparatus (Corbett Research, Biolabo, Archamps, France) and the SYBR Green I master mix (Roche Diagnostics, Mannheim, Germany). For each sample, the relative amount of Aurora A and GAPDH transcripts was calculated from the standard curves using the RotorGene software.

### Cell culture

The RCC4 cells come from ECACC [24] and 786-O and HeLa from ATCC. HeLa and RCC4 cells were maintained in DMEM medium and 786-O in RPMI medium, supplemented with 10% foetal bovine serum and antibiotics at 37^°^C in a humidified 5% CO_2_ atmosphere. Cells were transfected with the pCMV-VHL_213_ vector kindly provided by Dr Buchberger (Heidelberg, Germany) using JetPRIME^TM^ as recommended by the manufacturer (Polyplus, Illkirch, France). Cells were lysed in EB buffer (20 mM Tris-HCl pH 7.5, 100 mM NaCl, 5 mM MgCl_2,_ 10% glycerol, 0.5 mM DTT, 0.2% NP40, 20 mM β-glycerophosphate, 1 mM NaF, 1 mM AEBSF) 24 hours after transfection.

### Immunoprecipitation assays

Seventy µl of Dynabeads coupled to Protein G (Invitrogen, Saint-Aubin, France) were equilibrated with 500 µl of 0.5M acetate buffer pH 5.5 and incubated at 4°C with the anti-pVHL antibody or control IgG for 2 hours, and then washed twice with 500 µl PBS. Beads were then incubated at 4°C with 300 µg of HeLa or RCC4 protein extract or with an extract of 786-0 cells previously transfected for 24 hours with pCMV-VHL-213 during 2 hours. Beads were washed once in 500 µl of 0.5% NaCl and five times with 500 µl TBST (50 mM Tris-HCl, pH 7.5, 150mM NaCl, 0.05% Tween-20). Bound proteins were eluted in 10 µl of 2X Laemmli sample buffer, separated on a 12.5% SDS polyacrylamide gel and transferred to nitrocellulose membranes. Membranes were incubated with the anti-pVHL (1:100) or anti-Aurora-A antibody (1:200).

### Protein purification

Recombinant Aurora-A-(His)_6_, pVHL_213_-(His)_6_ and pVHL _213_S72A-(His)_6_ were produced in the *Escherichia coli* strain BL21(DE3) pLysS. Bacteria were lysed in IMAC 5 buffer (20 mM Tris-HCl, pH 7.5; 500 mM NaCl; 10% glycerol, and 5 mM imidazole) containing 1 mg/ml lysozyme and 1 mM PMSF for 1 h at 4 °C. After centrifugation at 12,000g at 4 °C (JA-20 rotor, Beckman Instruments) for 30 min, supernatants were filtered and proteins purified by Ni-NTA-agarose affinity chromatography following the manufacturer’s instructions (Qiagen, Courtaboeuf, France), as previously described [25]. The concentration of the eluted His-tagged protein fractions was determined according to the Bradford method and their purity was assessed by electrophoresis on 12.5% SDS gels.

### Protein Kinase Assay

Two µg of Aurora-A-(His) 6 were incubated in 15 µl of kinase buffer (50 mM Tris-HCl, pH 7.5; 25 mM NaCl; 1 mM dithiothreitol; 10 mM MgCl_2_) in the presence of 5 µCi [γ -32P] ATP at 3,000 Ci/mmol and 2 µg pVHL_213_-(His)_6_ or pVHL _213_S/A72-(His)_6_ at 30°C for 10 min. Reactions were stopped by addition of 5 µl of denaturing 5X Laemmli sample buffer and proteins were separated on 12.5% SDS-PAGE gels. Incorporation of ^32^P was analysed by autoradiography.

### Western blot analysis

Frozen tissues were lysed in RIPA buffer (50 mM Tris-HCl, pH 7.4, 1% NP-40, 0.5% sodium deoxycholate, 150 mM sodium chloride, 1 mM EDTA, 1 mM sodium fluoride, 1 mM AEBSF, 10 µg/ml aprotinin, 10 µg/ml leupeptin, 1 mM sodium orthovanadate) and centrifuged at 9000g for 10 min. Bradford assays were performed to determine the protein concentrations in the supernatants. Samples (50 µg of total proteins) were separated on 15% polyacrylamide gels and transferred onto nitrocellulose membranes. Membranes were washed with TBST, saturated with 5% low fat milk in TBST at room temperature for 2 hr and then incubated with the primary antibodies in 2.5% low fat milk in TBST at 4°C overnight. Antibody binding was detected with the appropriate horseradish peroxidase conjugated secondary antibody (1:15,000 in TBST-2.5% BSA at RT for 1h) and the Western Blot chemiluminescence Super Signal kit (Pierce, Rockford, IL). Sample loading was controlled using an anti-Actin polyclonal antibody (1:500).

### Immunohistochemistry

Five-µm sections of formalin-fixed paraffin-embedded tissue samples mounted on glass slides were dewaxed with xylene and rehydrated through a gradient series of ethanol. They were then heated at 100°C in hot 0.01 mM citrate buffer pH 8 for 40 min, left to cool down for 20 minutes and washed in PBS/0.1% Tween for 5 min. Endogenous peroxidase was quenched in 3% hydrogen peroxide for 10 min. Slides were then washed in PBS/0.1% Tween for 5 min and incubated with the primary antibodies against Aurora-A (1:1000) and pVHL (1:100). Antibody binding was revealed with HRP (horseradish peroxidase)-labelled polymer conjugated to secondary antibodies (Envision™ + Dual Link System-HRP, DAKO) using diaminobenzidine as chromogen (Sigma-Aldrich). Antibody staining was assessed using a Leica™ DMRXA microscope equipped with a CoolSnapsHQ camera (Photometrics™).

### Immunofluorescence

After blocking in PBS/5% BSA, Hela cells and RCC4 cells cultivated and fixed onto glass slides were incubated at room temperature with the mouse anti-pVHL monoclonal antibody (1:200) and/or the rabbit anti-Aurora-A (1:200) antibody diluted in PBS/1% BSA for 1 h. After two washings with PBS, slides were incubated with Alexa Fluor® 546-conjugated anti-rabbit and Alexa Fluor® 488 donkey anti-mouse secondary antibodies (1:15000 in PBS/1% BSA) at room temperature for one hour, then washed in PBS and mounted in Vectashield^®^ containing 1µg/ml DAPI. Imaging was performed on a Leica DM 5500 confocal microscope using a X63 HCX PLAN-APO-ON 1.4 oil immersion objective lens (MRic microscopy platform). Sequential images were captured at a single focal plane using appropriate laser settings for the detection of DNA (TO-PRO®-3), Aurora-A and VHL. Image processing was performed with the ImageJ 1.4 software.

#### Statistical analysis

Analyses were performed using the GraphPad software. The non parametric Mann–Withney test was used to evaluate the statistical relationship between the expression of Aurora-A protein and the VHL status (wt/wt or VHL mut/del) as well as with the Fuhrman grade of the tumour. For all analyses, differences were considered to be significant when p < 0.05. A linear regression analysis was used to study the relation between the expression of Aurora-A protein and the expression of cytoplasmic VHL protein leading to the determination of the Pearson coefficient.

## Results

### VHL status of tumour samples

First we determined the VHL status (wild type – VHLwt/wt or presence of mutations/deletions – VHLmut/del) of the surgical ccRCC samples from a cohort of 30 patients. Ten specimens (33% of the series) were VHLwt/wt. In the remaining 20 tumours (67%) with VHLmut/del, deletion of the gene in one allele was associated with mutations in the other allele (VHLmut/del). Among the 20 samples which were characterised as VHLmut /del, 10 samples were mutated in exon 1, 6 samples in exon 2 and 4 samples in exon 3. They were mainly stop insertions, frame shift or missense mutations and 50% of them were in exon 1 and 13% in exon 3. Overall, 64% of patients had a Fuhrman grade 3 to 4 tumour. *VHL*wt/wt ccRCCs mostly had a high Fuhrman grade score (80%), while VHLmut/del tumours were equally distributed among grades 2 to 4 (Table 1).

**Table 1:** Fuhrman grade and *VHL* status of the 30 clear cell renal cell carcinoma (ccRCC) specimens under study.

**Fuhrman grade**	**1**	**2**	**3**	**4**
**VHLwt/wt % (nb)^a^**	**3 (1)**	**3 (1)**	**10 (3)**	**17 (5)**
**VHLmut/del % (nb)**	**0**	**33 (10)**	**23 (7)**	**10 (3)**

a *(nb) : number of samples*

### The expression level of Aurora-A transcripts in ccRCCs is correlated with the Fuhrman grade and the VHL status

**Figure 1:**
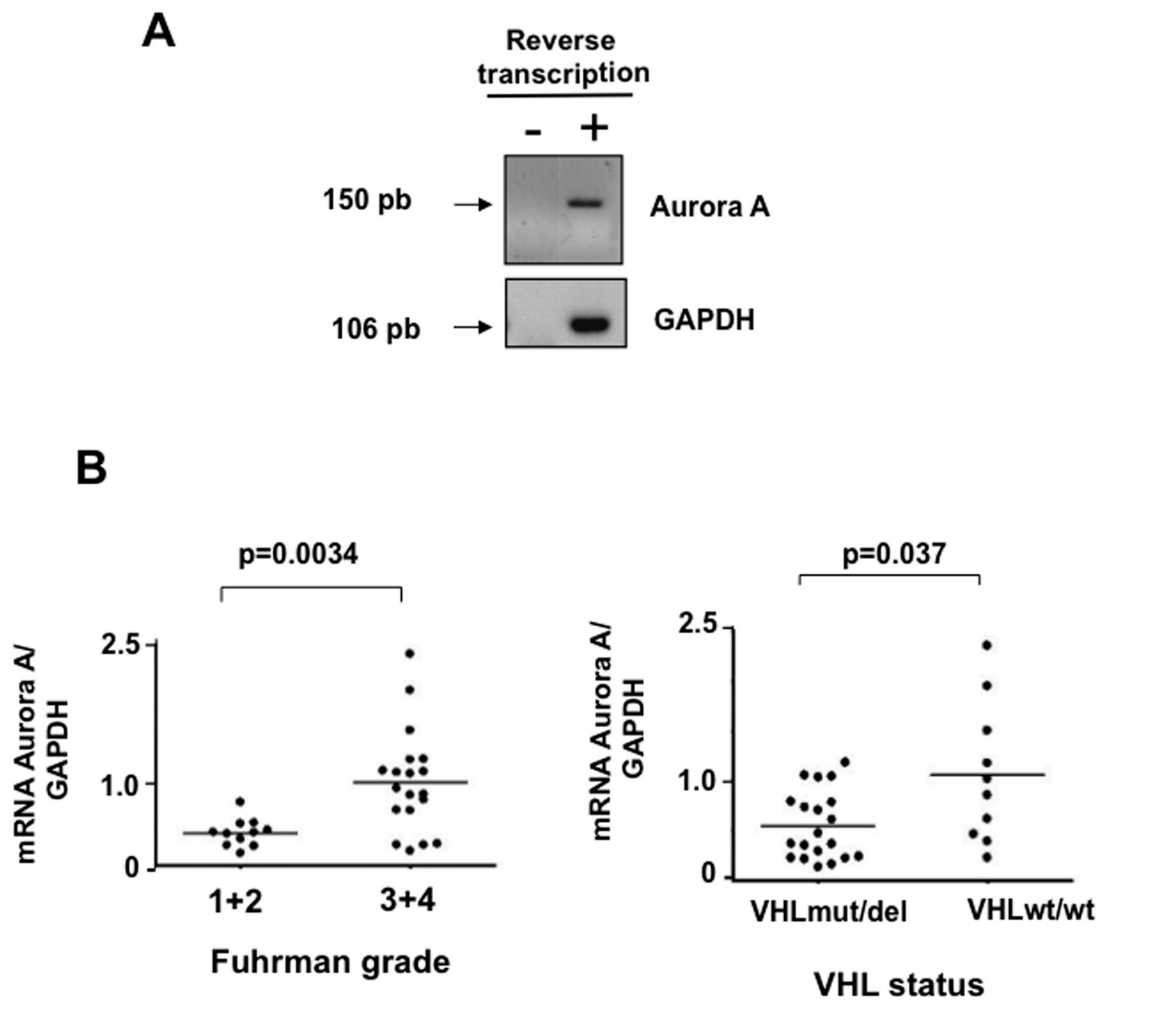
Relative expression of the Aurora-A kinase in ccRCC and matching healthy tissue samples. **A-**Expression of Aurora-A in tumours and neighbouring healthy tissues. Total RNAs extracted from tumour tissues were reverse transcribed and Aurora-A expression was then analysed by PCR using specific Aurora-A primers that produced an amplicon of 150 bp. Expression values were normalized to GAPDH levels. **B**-Aurora-A transcript levels are correlated with the tumour Fuhrman grade and *VHL* status. Quantitative RT-PCR data were normalized to GAPDH and then their distribution according to the tumour grade and VHL status was analysed using the Mann-Whitney test. The Aurora-A transcription level of each tumour is symbolized by a dot and the median by a horizontal line.

We then measured Aurora-A expression in the 30 surgical ccRCC samples (T) by quantitative real-time PCR (Figure 1A). The Aurora-A transcript levels were significantly higher in Fuhrman grade 3 and 4 than in grade 1 and 2 tumours (p=0.0034) and in VHLwt/wt than in VHL mut/del ccRCC samples (p=0.037) (Figure 1B).

### Aurora-A and VHL protein expression in matching ccRCC and healthy kidney samples

**Figure 2:**
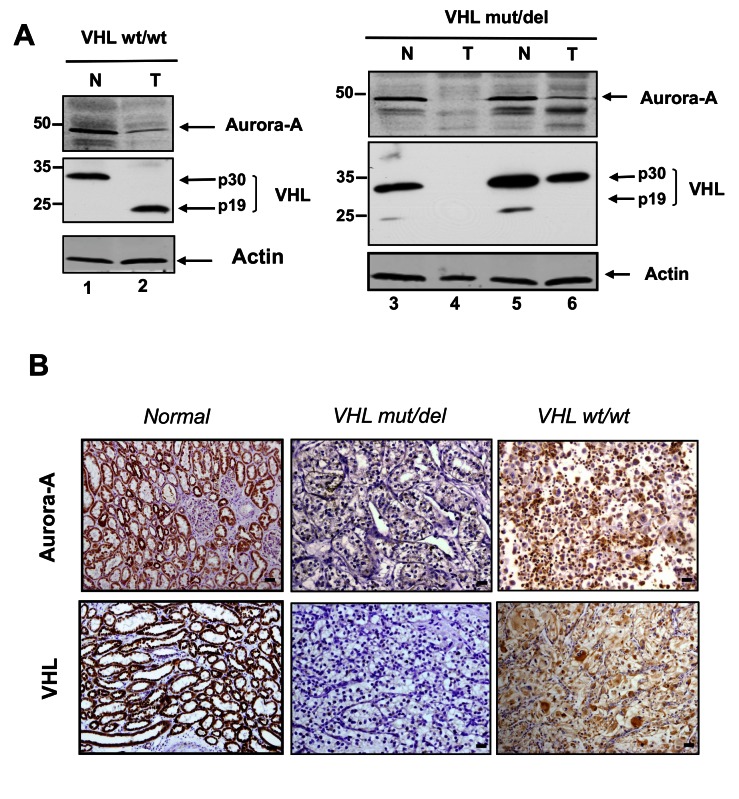
Detection of Aurora-A and VHL in healthy and tumour tissues. A-Western blot analysis of protein extracts from ccRCC and matching healthy tissue samples. Total proteins from tumour (T) and healthy (N) tissue samples were extracted using the RIPA buffer and analysed by western blotting with the anti-Aurora-A polyclonal antibody (1:200) and the anti-pVHL monoclonal antibody (1:500). β-actin was used as loading control. B-Immunohistochemical analysis of Aurora-A and VHL expression in ccRCC and matching healthy tissue samples. Tissues samples were prepared for immunohistochemistry as described in Materials and Methods and analysed using the polyclonal antibody against Aurora-A (1:100) and the monoclonal antibody against pVHL (1:200). Microphotographs were acquired with a Leica™ DMRXA microscope equipped with a CoolSnapsHQ camera (Photometrics™) (bar 10µm).

We investigated VHL (pVHL30 and pVHL19) and Aurora-A protein expression by western blot analysis using protein extracts from ccRCC samples (T) and matching neighbouring healthy tissues (N) from 2 VHL wt/wt samples and 9 VHL mut/del samples (Figure 2A). In all normal samples, the pVHL30 isoform was predominant, whereas pVHL19 was weakly or not expressed (Figure 2A: lanes 1, 3 and 5). In tumour tissues, the expression of both VHL isoforms was very variable and depended on the *VHL* status. VHLwt/wt tumour samples expressed only pVHL19 (Figure 2A: lane 2). Conversely, in VHLmut/del tumours no pVHL or only the pVHL30 isoform could be detected. Aurora-A was detected at the predicted molecular weight of 42 kDa in all normal tissue samples (Figure 2A: lanes 1, 3 and 5) and in most tumour tissues, albeit at lower level (Figure 2A: lanes 2, 6). In few tumour samples, Aurora-A could not be detected (Figure 2A, lane 4).

**Figure 3:**
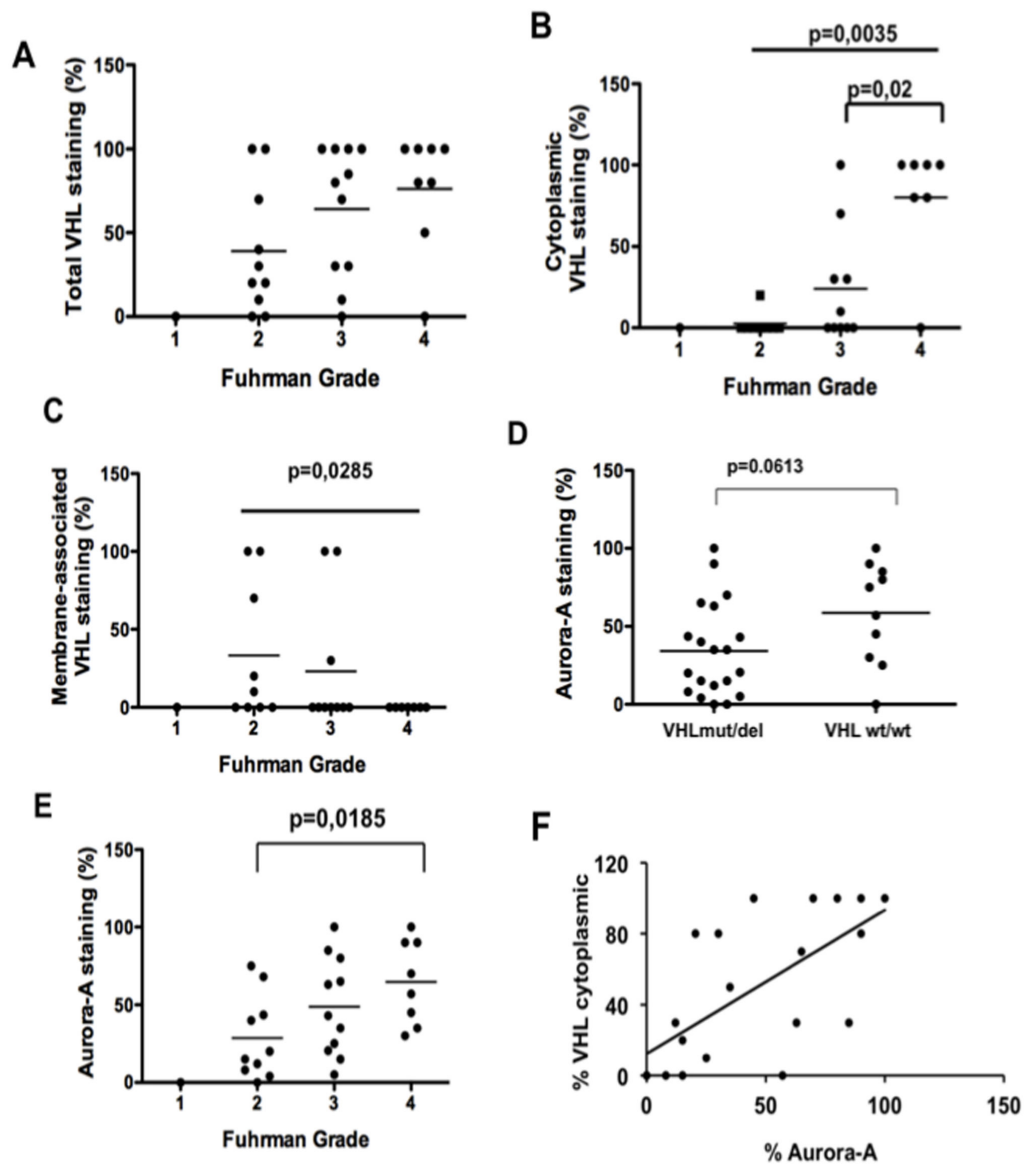
Statistical analysis of Aurora-A and pVHL levels and localization in ccRCCs according to their Fuhrman grade and *VHL* status. pVHL expression level (A) and localization (B and C) in ccRCCs were analysed relative to the Fuhrman grade of the tumours. Aurora-A expression level was assessed relative to the VHL status (D) and Fuhrman grade (E) of the tumours. Aurora-A expression according to the VHL cytoplasmic localization was also evaluated (F). The non-parametric Mann-Whitney test was used to evaluate the statistical relationships between the expression of Aurora-A and pVHL and the tumour parameters.

We then analysed the localization of pVHL and Aurora-A by IHC (Figure 2B). In normal renal tissues, VHL and Aurora-A were homogeneously expressed in the epithelial cells of proximal and distal tubules (Figure 2B: left panels). In tumours the tissue architecture was dramatically disorganized and immunoreactivity towards Aurora-A and pVHL was variable (Figure 2B). All VHLwt/wt samples expressed pVHL, while four of the VHLmut/del tumours did not (data not shown), in agreement with the western blot analysis. The intensity of pVHL staining was not correlated with the tumour grade (Figure 3A), whereas its localization differed with the tumour grade. In high grade tumours, pVHL expression was predominantly cytoplasmic (grade 4 vs. grade 2: p=0.0035; grade 4 vs. grade 3: p=0.002; Figure 3B), while in low grade tumours, pVHL was mostly membrane-associated (grade 2 vs. grade 4: p=0.0285; Figure 3C).

The intensity of Aurora-A staining was correlated with the tumour grade (grade 4 *vs.* grade 2: p=0.0185) (Figure 3E), but not with the VHL status (p=0.0613) (Figure 3D), differently from what observed for the transcript level. This discrepancy could be due to the limited number of patients examined in our study (n=30). Moreover, Aurora-A staining was mainly observed in cells with pVHL cytoplasmic expression (Figure 3F, Pearson coefficient 0.658).

### Aurora–A interacts with and phosphorylates pVHL

**Figure 4:**
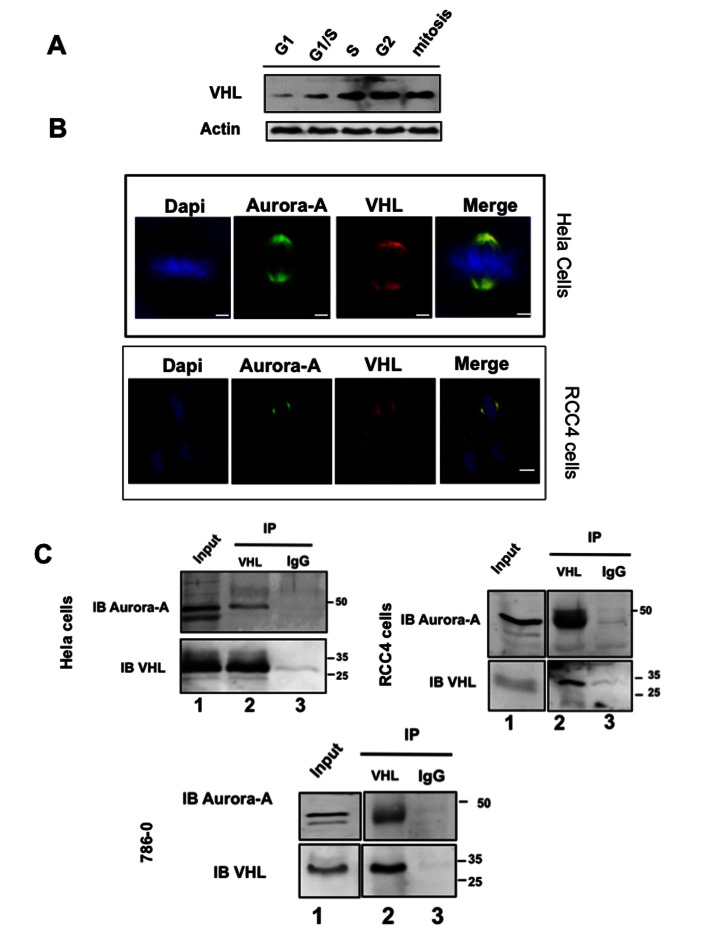
pVHL interacts with Aurora-A. A-HeLa cells were synchronized as described in Materials and Methods. Twenty µg of protein extracts were separated on a poly-acrylamide gel and VHL expression was assessed with the monoclonal anti-pVHL antibody (1:200). Loading was controlled with the anti-Actin antibody (1:200). B-In HeLa and RCC4 cells, Aurora-A and VHL co-localize at the centrosome and at the mitotic spindle extremities (anti-Aurora-A antibody, 1:200 and anti-pVHL antibody, 1:200). C-HeLa, RCC4 and 786-0 cell protein extracts were immunoprecipitated with the anti-pVHL polyclonal antibody (lane 2) or with the IgG (lane 3, negative control). Aurora-A and pVHL were both detected in the immunoprecipitates using a polyclonal antibody against Aurora-A (1:200) (upper panel) and a monoclonal antibody against pVHL (1:200) (lower panel). Lane 1 was corresponding to the total cell extract.

Aurora-A and pVHL co-expression in tumour cells and their implication in regulating the microtubule network and cell cycle progression suggested that they may interact. To test this hypothesis, first we studied their expression and localization during the cell cycle in HeLa cells in which pVHL30 is endogenously expressed. In these cells, pVHL30 expression varied during the cell cycle, reaching a maximum in the G2 phase (Figure 4A). As it was previously shown that Aurora-A expression and activity are cell cycle-regulated and that Aurora-A is mainly associated with centrosomes from the end of S phase to the beginning of the G1 phase [26], we assessed simultaneously the localization of Aurora-A and pVHL by double immunofluorescence. In HeLa cells, as well as in RCC4 cells, both proteins co-localized at the centrosome and at the mitotic spindle extremities (Figure 4B).

**Figure 5:**
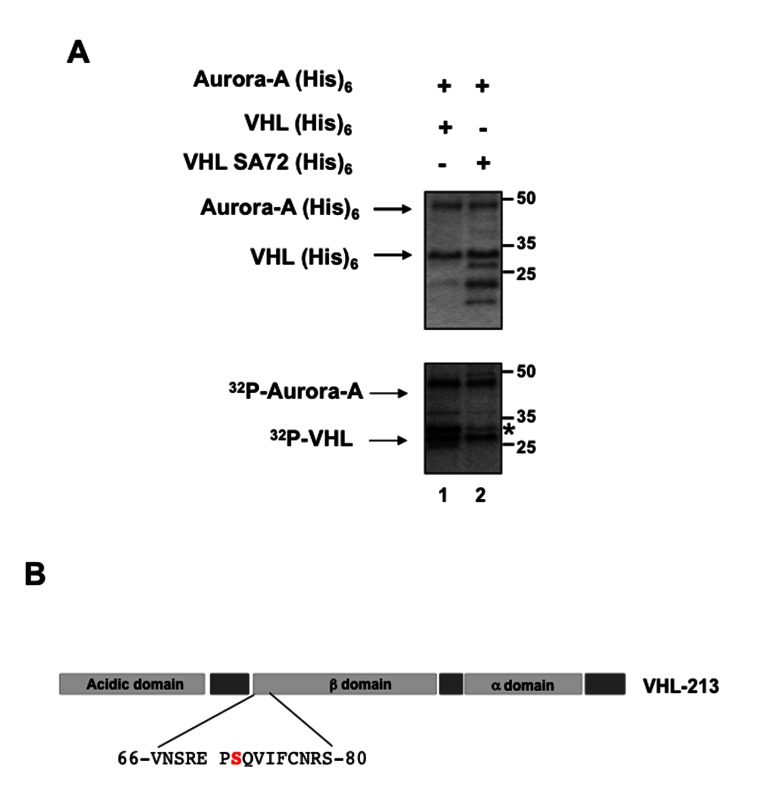
pVHL is phosphorylated on Ser72 by Aurora-A *in vitro*. A- Purified recombinant 6xHis-tagged pVHL30 [VHL(His)_6_] (lane 1) or mutant 6xHis-tagged pVHL30 S72A [VHLSA72(His)_6_] ( (lane 2) were incubated in the presence of [γ-^32^P] ATP and recombinant Aurora-A(His) 6. Samples were separated by SDS-PAGE and the gel was stained with Coomassie Blue (higher panel). pVHL phosphorylation status was analysed by autoradiography (lower panel). The asterisk (*) indicate the pVHL protein. B-Localization and sequence of the putative Aurora-A phosphorylation site on pVHL.

Then, we investigated whether Aurora-A and pVHL interacted by immunoprecipitation assay using an anti-VHL antibody and total proteins extracts from HeLa cells that had been transfected with the pCMV-hVHL_213_ plasmid to over-express pVHL30. The interaction was also assayed by performing the immunoprecipitation on protein extracts of two different kidney cell lines (RCC4 and 786-0) (Figure 4C). As Aurora-A was co-immunoprecipitated with pVHL in all three cell lines (Figure 4C), we then asked whether pVHL was a phosphorylation substrate of Aurora-A. We thus incubated affinity-purified recombinant Aurora-A(His)_6_ with recombinant pVHL-213(His)_6_ in the presence of [γ-^32^P] ATP (Figure 5). ^32^P was incorporated in VHL in the presence of Aurora-A (Figure 5: lane 1) and mass spectrometry analysis of ^32^P-VHL allowed identifying Ser72 as the most phosphorylated amino acid. Three different productions and purifications of pVHL and Aurora-A proteins were done and used in two independent kinase assays before identifying the phosphorylation site. Accordingly, the sequence which includes Ser72 (…SREPS*QVIF…) matched the putative Aurora-A consensus phosphorylation site [(K/R) xx(S/T)] (Figure 5). Mutation of Ser72 into alanine (VHL SA72) greatly diminished phosphorylation of pVHL by Aurora-A (Figure 5, lane 2).

## Discussion

Inherited and sporadic forms of ccRCC account for 85% of renal cancers and loss of the *VHL* tumour suppressor gene is involved in 70% of sporadic ccRCC. In addition to genetic mutations, ccRCC is mainly associated with genetic instability that could be caused by abrogation of the cell cycle mitotic spindle checkpoint and may involve the centrosomal kinase Aurora-A, which regulates centrosome stability and thus influences cell cycle progression [27].

In the present study, we determined the relationship between Aurora-A and pVHL in ccRCC. Higher levels of Aurora-A transcripts were significantly associated with high Fuhrman grade ccRCC samples (grades 3 and 4) and with the VHLwt/wt status. Similarly, Aurora-A protein expression both at the cell membrane and in the cytoplasm was significantly higher in grade 3-4 tumours and tended to be more elevated in VHLwt/wt tumours. These results suggest that low levels of Aurora A could be correlated with the presence of non-functional pVHL.

Previous studies in ductal breast cancer showed that Aurora-A over-expression was independent of the tumour histopathological type and was not correlated with tumour size and lymph node metastases [28]. Conversely, other authors showed that Aurora-A alterations were associated with poor prognosis in gastric cancers and high grade/late stage in breast and bladder cancers [29] [30]. An exhaustive analysis of Aurora-A expression in human RCC revealed no significant association with major pathologic factors [21]; however, in this study the relation with the VHL status was not assessed.

**Figure 6:**
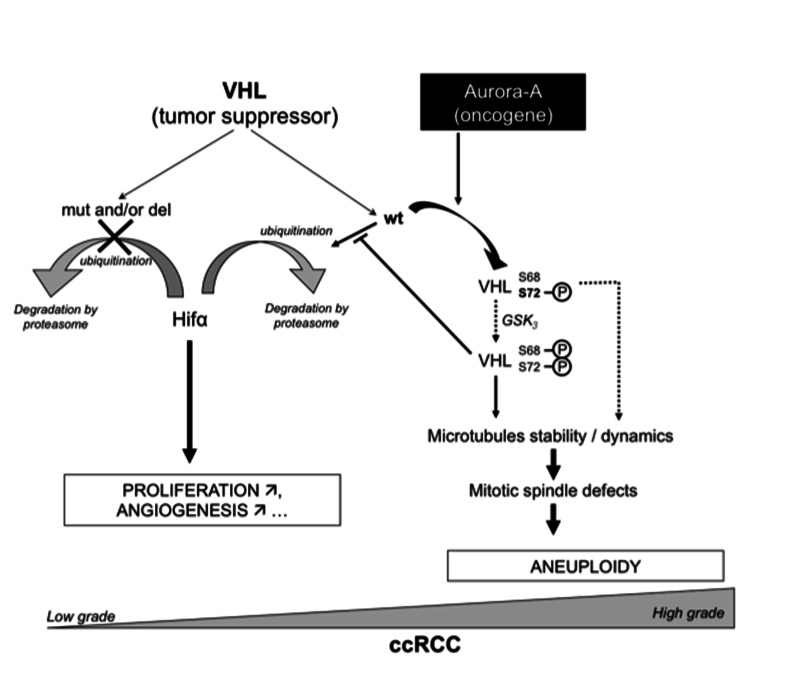
Putative function of the interaction between VHL and Aurora-A in the ccRCC.

About 80% of the VHLwt/wt tumours included in our study were grade 3-4, while only 50% of VHLmut/del tumours were classified as grade 3 and 4. It has been reported that the outcome of patients with wild-type *VHL* is significantly worse both in terms of progression free-survival (PFS) and RCC-specific survival (RCC-SS), whereas VHL alterations appear to be associated with a more favourable outcome [22]. pVHL expression in our series was very heterogeneous as previously reported [3] [31] and not correlated with the tumour grade. However, pVHL cytoplasmic localization was associated with grade 3-4 tumours and with higher Aurora-A expression, suggesting that Aurora-A oncogenic action might also involve interaction with pVHL to deregulate its tumour suppressor function. Indeed, we further show that pVHL30 interacts with and is phosphorylated by Aurora-A at Ser 72 in the β-domain of pVHL. Several putative phosphorylation sites on pVHL have been identified by mass spectrometry. Three of these amino acids are located in the acidic region of pVHL (Ser33, Ser38 and Ser48) and three others are in the β-domain (Ser68, Ser72 and Ser111). Ser111 of pVHL is a physiological target of Checkpoint kinase 2 (CHK2) in response to DNA damage. Phosphorylation of Ser68 by glycogen synthase kinase 3 (GSK3) negatively regulates pVHL-mediated microtubule stabilization [18,32]. GSK-3 regulation requires a priming phosphorylation on S72, which *in vitro* can be accomplished by Casein kinase 1 (CK1). Our data identify Aurora A as the putative kinase that might carry out the priming phosphorylation on Ser 72 *in vivo*. As pVHL was identified as a microtubule-associated protein which prevents microtubules depolymerisation, we therefore speculated that the phosphorylation of VHL by Aurora-A would modulate the stability and/or dynamics of the microtubules. Dysregulation of such coordination may hence be responsible of mitotic defects leading to aneuploidy, which contributes to tumorigenesis (Figure 6). In conclusion, the present study describes for the first time the relationship between pVHL and the oncogenic Aurora-A protein kinase. Even though the expression levels of both proteins might not be significantly correlated with tumour progression, we hypothesize that the functional interaction between Aurora-A and pVHL may participate in cell cycle regulation and in tumour initiation/progression through signalling pathway(s) that need to be further investigated.

## References

[1] Seizinger BR, Rouleau GA, Ozelius LJ, Lane AH, Farmer GE et al. (1988) Von Hippel-Lindau disease maps to the region of chromosome 3 associated with renal cell carcinoma. Nature 332: 268-269. doi:10.1038/332268a0. PubMed: 2894613. 2894613 10.1038/332268a0

[2] Crossey PA, Richards FM, Foster K, Green JS, Prowse A et al. (1994) Identification of intragenic mutations in the von Hippel-Lindau disease tumour suppressor gene and correlation with disease phenotype. Hum Mol Genet 3: 1303-1308. doi:10.1093/hmg/3.8.1303. PubMed: 7987306. 7987306 10.1093/hmg/3.8.1303

[3] Kovacs G, Akhtar M, Beckwith BJ, Bugert P, Cooper CS et al. (1997) The Heidelberg classification of renal cell tumours. J Pathol 183: 131-133. doi:10.1002/(SICI)1096-9896(199710)183:2. PubMed: 9390023. 9390023 10.1002/(SICI)1096-9896(199710)183:2<131::AID-PATH931>3.0.CO;2-G

[4] Chen F, Kishida T, Duh FM, Renbaum P, Orcutt ML et al. (1995) Suppression of growth of renal carcinoma cells by the von Hippel-Lindau tumor suppressor gene. Cancer Res 55: 4804-4807. PubMed: 7585510. 7585510

[5] Foster K, Crossey PA, Cairns P, Hetherington JW, Richards FM et al. (1994) Molecular genetic investigation of sporadic renal cell carcinoma: analysis of allele loss on chromosomes 3p, 5q, 11p, 17 and 22. Br J Cancer 69: 230-234. doi:10.1038/bjc.1994.44. PubMed: 8297719. 8297719 10.1038/bjc.1994.44 PMC1968700

[6] Kaelin WG Jr. (2002) Molecular basis of the VHL hereditary cancer syndrome. Nat Rev Cancer 2: 673-682. doi:10.1038/nrc885. PubMed: 12209156. 12209156 10.1038/nrc885

[7] Kim WY, Kaelin WG (2004) Role of VHL gene mutation in human cancer. J Clin Oncol 22: 4991-5004. doi:10.1200/JCO.2004.05.061. PubMed: 15611513. 15611513 10.1200/JCO.2004.05.061

[8] Pause A, Lee S, Lonergan KM, Klausner RD (1998) The von Hippel-Lindau tumor suppressor gene is required for cell cycle exit upon serum withdrawal. Proc Natl Acad Sci U S A 95: 993-998. doi:10.1073/pnas.95.3.993. PubMed: 9448273. 9448273 10.1073/pnas.95.3.993 PMC18649

[9] Seagroves T, Johnson RS (2002) Two HIFs may be better than one. Cancer Cell 1: 211-213. doi:10.1016/S1535-6108(02)00048-X. PubMed: 12086854. 12086854 10.1016/s1535-6108(02)00048-x

[10] Hergovich A, Lisztwan J, Barry R, Ballschmieter P, Krek W (2003) Regulation of microtubule stability by the von Hippel-Lindau tumour suppressor protein pVHL. Nat Cell Biol 5: 64-70. doi:10.1038/ncb899. PubMed: 12510195. 12510195 10.1038/ncb899

[11] Thoma CR, Toso A, Gutbrodt KL, Reggi SP, Frew IJ et al. (2009) VHL loss causes spindle misorientation and chromosome instability. Nat Cell Biol 11: 994-1001. doi:10.1038/ncb1912. PubMed: 19620968. 19620968 10.1038/ncb1912

[12] Bischoff JR, Anderson L, Zhu Y, Mossie K, Ng L et al. (1998) A homologue of Drosophila aurora kinase is oncogenic and amplified in human colorectal cancers. EMBO J 17: 3052-3065. doi:10.1093/emboj/17.11.3052. PubMed: 9606188. 9606188 10.1093/emboj/17.11.3052 PMC1170645

[13] Arlot-Bonnemains Y, Klotzbucher A, Giet R, Uzbekov R, Bihan R et al. (2001) Identification of a functional destruction box in the Xenopus laevis aurora-A kinase pEg2. FEBS Lett 508: 149-152. doi:10.1016/S0014-5793(01)03048-4. PubMed: 11707286. 11707286 10.1016/s0014-5793(01)03048-4

[14] Royce ME, Xia W, Sahin AA, Katayama H, Johnston DA et al. (2004) STK15/Aurora-A expression in primary breast tumors is correlated with nuclear grade but not with prognosis. Cancer 100: 12-19. doi:10.1002/cncr.11879. PubMed: 14692019. 14692019 10.1002/cncr.11879

[15] Buschhorn HM, Klein RR, Chambers SM, Hardy MC, Green S et al. (2005) Aurora-A over-expression in high-grade PIN lesions and prostate cancer. Prostate 64: 341-346. doi:10.1002/pros.20247. PubMed: 15754349. 15754349 10.1002/pros.20247

[16] Ulisse S, Baldini E, Toller M, Delcros JG, Guého A et al. (2007) Transforming acidic coiled-coil 3 and Aurora-A interact in human thyrocytes and their expression is deregulated in thyroid cancer tissues. Endocr Relat Cancer 14: 827-837. doi:10.1677/ERC-07-0053. PubMed: 17914111. 17914111 10.1677/ERC-07-0053 PMC2216418

[17] Ulisse S, Delcros JG, Baldini E, Toller M, Curcio F et al. (2006) Expression of Aurora kinases in human thyroid carcinoma cell lines and tissues. Int J Cancer 119: 275-282. doi:10.1002/ijc.21842. PubMed: 16477625. 16477625 10.1002/ijc.21842

[18] Ampofo E, Kietzmann T, Zimmer A, Jakupovic M, Montenarh M et al. (2010) Phosphorylation of the von Hippel-Lindau protein (VHL) by protein kinase CK2 reduces its protein stability and affects p53 and HIF-1alpha mediated transcription. Int J Biochem Cell Biol 42: 1729-1735. doi:10.1016/j.biocel.2010.07.008. PubMed: 20637892. 20637892 10.1016/j.biocel.2010.07.008

[19] Motzer RJ, Bander NH, Nanus DM (1996) Renal-cell carcinoma. N Engl J Med 335: 865-875. doi:10.1056/NEJM199609193351207. PubMed: 8778606. 8778606 10.1056/NEJM199609193351207

[20] Ehara H, Yokoi S, Tamaki M, Nishino Y, Takahashi Y et al. (2003) Expression of mitotic Aurora/Ipl1p-related kinases in renal cell carcinomas: an immunohistochemical study. Urol Res 31: 382-386. doi:10.1007/s00240-003-0354-x. PubMed: 13680024. 13680024 10.1007/s00240-003-0354-x

[21] Kurahashi T, Miyake H, Hara I, Fujisawa M (2007) Significance of Aurora-A expression in renal cell carcinoma. Urol Oncol 25: 128-133. doi:10.1016/j.urolonc.2006.06.001. PubMed: 17349527. 17349527 10.1016/j.urolonc.2006.06.001

[22] Patard JJ, Rioux-Leclercq N, Masson D, Zerrouki S, Jouan F et al. (2009) Absence of VHL gene alteration and high VEGF expression are associated with tumour aggressiveness and poor survival of renal-cell carcinoma. Br J Cancer 101: 1417-1424. doi:10.1038/sj.bjc.6605298. PubMed: 19755989. 19755989 10.1038/sj.bjc.6605298 PMC2768461

[23] Patard JJ (2009) Incidental renal tumours. Curr Opin Urol 19: 454-458. doi:10.1097/MOU.0b013e32832f0ccd. PubMed: 19571758. 19571758 10.1097/MOU.0b013e32832f0ccd

[24] Maxwell PH, Wiesener MS, Chang GW, Clifford SC, Vaux EC et al. (1999) The tumour suppressor protein VHL targets hypoxia-inducible factors for oxygen-dependent proteolysis. Nature 399: 271-275. doi:10.1038/20459. PubMed: 10353251. 10353251 10.1038/20459

[25] Klotzbucher A, Pascreau G, Prigent C, Arlot-Bonnemains Y (2002) A Method for Analyzing the Ubiquitination and Degradation of Aurora-A. Biol Proced Online 4: 62-69. doi:10.1251/bpo35. PubMed: 12734567. 12734567 10.1251/bpo35 PMC145558

[26] Kimura M, [!(surname)!], Hattori T, Sumi N, Yoshioka T, Todokoro K, Okano Y (1997) Cell cycle-dependent expression and spindle pole localization of a novel human protein kinase, Aik, related to Aurora of Drosophila and yeast Ipl1. J Biol Chem 272(21): 13766-13771. doi:10.1074/jbc.272.21.13766. PubMed: 9153231. 9153231 10.1074/jbc.272.21.13766

[27] Bodmer D, Eleveld M, Ligtenberg M, Weterman M, van der Meijden A et al. (2002) Cytogenetic and molecular analysis of early stage renal cell carcinomas in a family with a translocation (2;3)(q35;q21). Cancer Genet Cytogenet 134: 6-12. doi:10.1016/S0165-4608(01)00585-4. PubMed: 11996788. 11996788 10.1016/s0165-4608(01)00585-4

[28] Hoque A, Carter J, Xia W, Hung MC, Sahin AA et al. (2003) Loss of aurora A/STK15/BTAK overexpression correlates with transition of in situ to invasive ductal carcinoma of the breast. Cancer epidemiology, biomarkers & prevention : a publication of the American Association for Cancer Research, cosponsored by the American Society of Preventive Oncology 12: 1518-1522. 14693746

[29] Lei Y, Yan S, Ming-De L, [!(surname)!], Rui-Fa H (2011) Prognostic significance of Aurora-A expression in human bladder cancer. Acta histochemica 113: 514-518. 20598352 10.1016/j.acthis.2010.05.004

[30] Bufo P, Sanguedolce F, Tortorella S, Cormio L, Carrieri G et al. (2010) Expression of mitotic kinases phospho-aurora A and aurora B correlates with clinical and pathological parameters in bladder neoplasms. Histol Histopathol 25: 1371-1377. PubMed: 20865660. 20865660 10.14670/HH-25.1371

[31] Thoenes W, Storkel S, Rumpelt HJ (1986) Histopathology and classification of renal cell tumors (adenomas, oncocytomas and carcinomas). The basic cytological and histopathological elements and their use for diagnostics. Pathol Res Practice 181: 125-143. doi:10.1016/S0344-0338(86)80001-2. 10.1016/S0344-0338(86)80001-2 3737468

[32] Hergovich A, Lisztwan J, Thoma CR, Wirbelauer C, Barry RE et al. (2006) Priming-dependent phosphorylation and regulation of the tumor suppressor pVHL by glycogen synthase kinase 3. Mol Cell Biol 26: 5784-5796. doi:10.1128/MCB.00232-06. PubMed: 16847331. 16847331 10.1128/MCB.00232-06 PMC1592755

