# Correction: Identification of pVHL as a Novel Substrate for Aurora-A in Clear Cell Renal Cell Carcinoma (ccRCC)

**DOI:** 10.1371/annotation/d506055a-d16b-4c0c-aef6-ad39e91a3b8d

**Published:** 2014-01-03

**Authors:** Benedicte Martin, Franck Chesnel, Jean-Guy Delcros, Florence Jouan, Anne Couturier, Frederic Dugay, Xavier Le Goff, Jean-Jacques Patard, Patricia Fergelot, Cecile Vigneau, Nathalie Rioux-Leclerq, Yannick Arlot-Bonnemains

An error was introduced in the preparation of this article for publication. Figure 5 is not complete and not present in its proper place.

Please find a complete version of Figure 5 here: http://plosone.org/corrections/pone.0067071.g005.cn.tif

The Figure 5 legend is:

**pVHL is phosphorylated on Ser72 by Aurora-A in vitro**

A- Purified recombinant 6xHis-tagged pVHL30 [VHL(His)6] (lane 1) or mutant 6xHis-tagged pVHL30 S72A [VHLSA72(His)6] ( (lane 2) were incubated in the presence of [γ-32P] ATP and recombinant Aurora-A(His)6. Samples were separated by SDS-PAGE and the gel was stained with Coomassie Blue (higher panel). pVHL phosphorylation status was analysed by autoradiography (lower panel).The asterix (*) indicate the pVHL protein.

B- Localization and sequence of the putative Aurora-A phosphorylation site on pVHL.

